# Microenvironmental regulation of cancer cell metabolism: implications for experimental design and translational studies

**DOI:** 10.1242/dmm.035758

**Published:** 2018-08-07

**Authors:** Alexander Muir, Laura V. Danai, Matthew G. Vander Heiden

**Affiliations:** 1 Koch Institute for Integrative Cancer Research, Department of Biology, Massachusetts Institute of Technology, Cambridge, MA 02139, USA; 2 Department of Biochemistry and Molecular Biology, University of Massachusetts Amherst, Amherst, MA 01003, USA; 3 Dana-Farber Cancer Institute, Boston, MA 02115, USA

**Keywords:** Cancer, Cancer models, Metabolism, Microenvironment, Nutrient availability, Nutrient sensing

## Abstract

Cancers have an altered metabolism, and there is interest in understanding precisely how oncogenic transformation alters cellular metabolism and how these metabolic alterations can translate into therapeutic opportunities. Researchers are developing increasingly powerful experimental techniques to study cellular metabolism, and these techniques have allowed for the analysis of cancer cell metabolism, both in tumors and in *ex vivo* cancer models. These analyses show that, while factors intrinsic to cancer cells such as oncogenic mutations, alter cellular metabolism, cell-extrinsic microenvironmental factors also substantially contribute to the metabolic phenotype of cancer cells. These findings highlight that microenvironmental factors within the tumor, such as nutrient availability, physical properties of the extracellular matrix, and interactions with stromal cells, can influence the metabolic phenotype of cancer cells and might ultimately dictate the response to metabolically targeted therapies. In an effort to better understand and target cancer metabolism, this Review focuses on the experimental evidence that microenvironmental factors regulate tumor metabolism, and on the implications of these findings for choosing appropriate model systems and experimental approaches.

## Abstract (precis)

**Summary:** Here, we discuss the model systems for cancer metabolism studies and highlight their key strengths and pitfalls in addressing different questions, such as how the microenvironment affects cancer metabolism and response to therapy.

## Introduction

In cancer cells, signaling networks downstream of oncogenes and tumor suppressors affect metabolic pathways (Nagarajan et al., 2016). This recognition led to renewed interest in studying cancer as a metabolic disease. Indeed, mutations in genes that activate growth signaling pathways, such as rat sarcoma viral oncogene (*RAS*) (Kimmelman, 2015), phosphoinositide 3-kinase (*PI3K*; also known as *PIK3CA*) (Lien et al., 2016) and MYC proto-oncogene bHLH transcription factor (*MYC*) (Stine et al., 2015) have been shown to affect cellular metabolism. Mutations in tumor suppressors such as tumor protein p53 (*TP53*) (Vousden and Ryan, 2009), von Hippel-Lindau tumor suppressor (*VHL*) (Eales et al., 2016) and Kelch-like ECH-associated protein 1 (*KEAP1*) (Romero et al., 2017; Sayin et al., 2017) are also associated with altered cellular metabolism. Metabolic enzymes themselves can also harbor recurring mutations, and in select cases this can lead to altered enzyme function that contributes to cancer pathogenesis (Ye et al., 2018). These metabolic alterations can be functionally required for tumor growth (Luengo et al., 2017; Bobrovnikova-Marjon and Hurov, 2014), metastatic spread (Teoh and Lunt, 2018; Lehuede et al., 2016; Elia et al., 2018) and resistance to therapy (Zhao et al., 2013); thus, there is increasing interest in understanding and targeting the metabolic liabilities of cancers.

The development of more sophisticated cellular metabolism assays has increased our understanding of the functional effects of oncogenic alterations in metabolic enzyme regulation, activity and pathway use. Analytically, advances in nuclear magnetic resonance (NMR) (Markley et al., 2017), mass spectrometry (MS) (Jang et al., 2018; Kang et al., 2018) (see Glossary, Box 1) and computational resources allow researchers to detect and quantitate various metabolites. Coupled with stable isotope tracing (Box 1) and algorithms to infer metabolic pathway flux (Kang et al., 2018; Jang et al., 2018; Buescher et al., 2015; Kaushik and DeBerardinis, 2018; Niedenfuhr et al., 2015; Antoniewicz, 2018; Dai and Locasale, 2017), these tools enable researchers to assess how metabolism is altered in cancer. Other methods, including genetic or chemical screens that indirectly study cancer metabolism by perturbing the function of metabolic pathways, are also becoming increasingly powerful.

**Box 1. Glossary**

**Auxotrophic:** in the context of metabolism, auxotrophy refers to the inability of cells to synthesize a particular compound or metabolite and thus auxotrophic cells require access to that nutrient from extracellular sources.

**Entosis:** a cellular phenomenon that involves the engulfment and degradation of neighboring cells; a type of cell death.

**Exchange flux:** a phenomenon that can complicate analysis of stable isotope tracing experiments (see definition below) in which substrate-product interconversion leads to metabolite labeling regardless of the net direction of the reaction.

**Glutaminase (GLS):** an enzyme that catalyzes the first step in glutamine catabolism by converting glutamine to glutamate and ammonia. Can localize to the cytosol or mitochondria.

**Macropinocytosis:** a nonselective endocytic process by which bulk extracellular materials are internalized into cells.

**Mass spectrometry (MS):** a method usually coupled to gas chromatography or liquid chromatography and used to assess the chemical composition of metabolites in a given sample.

**Matrix effect:** a phenomenon that can complicate analysis of stable metabolites by MS (see definition below) in which compounds in the sample interfere with the ionization process necessary to detect a particular metabolite by MS. This can result in ionization suppression or enhancement and thus affect the accuracy of quantitative metabolite measurements. Compounds with high mass or polarity can cause matrix effects.

**Microtome:** a tool used to thinly slice tissues. Although typically used for producing thin tissue sections for histology, a microtome can be used to cut tumor slices that can be then cultured *in vitro* for several days.

**Nuclear magnetic resonance spectroscopy (NMR):** a method that can be used to assess metabolites in a given sample.

**Organoids:** a type of cell culturing method by which cancer cells [or other type(s) of cells] are embedded in a 3D matrix, such as collagen or basement membrane, with or without particular factors to promote growth as a 3D structure. These types of culturing methods better recapitulate the spatial organization and diversity of cells in tissues and tumors.

**Pooled genetic screens:** a tool to identify genes that contribute to a particular phenotype. Pooled genetic screens involve using multiple short hairpin RNAs (shRNAs) or single guide RNAs (sgRNAs) to silence or inhibit the expression of various genes in a target cell population, which may cover most of the genome, or a subset of genes such as metabolic enzymes. In this method, the genes for shRNAs or sgRNAs are integrated in the cell's genome. The depletion or enrichment for specific shRNAs or sgRNAs is measured with next generation sequencing techniques. A depleted or enriched shRNA or sgRNA that targets a particular gene provides information on selection for or against loss of that gene in a particular context.

**Spheroids:** a type of cell culturing method by which cells are grown in clusters or aggregates, typically without the addition of ECM or special factors to the culture medium. Spheroid culture can be promoted via a variety of manipulations including culturing cell clusters in low-attachment plates. This culturing method maintains some aspects of spatial architecture and cell-to-cell contact observed *in vivo*.

**Stable isotope tracing:** a method by which cells, tissues or animals are exposed to stable isotope-labeled nutrients, such as ^13^C-glucose, and the incorporation of the isotope-labeled atoms into various metabolites is measured using NMR or MS to infer metabolic pathway utilization.

Although these methodological advances now provide researchers with an unprecedented ability to assess cancer metabolism, deploying these techniques in the appropriate cancer model remains important. There are often trade-offs between the complexity of a given model and its tractability in metabolic experiments, and not every technique that assays cellular metabolism provides useful information in every model. Animal models in which tumors develop *in situ*, such as in genetically engineered mouse models of cancer, can recapitulate human cancer progression in some cases (Day et al., 2015; Gengenbacher et al., 2017). However, these models are inherently complex, and dissecting how the multiple cell types within a tumor interact within the microenvironment and with other tissues in the organism is a challenge. In contrast, *in vitro* culture models of cancer are experimentally tractable, but rely on studying cells in a context that is different from that of human tumors. In this Review, we discuss the common methods to study cellular metabolism and their application to various cancer models. We also highlight the experimental findings that inform how the tumor microenvironment influences cancer cell metabolism, and discuss the implications of these findings for choosing the appropriate models to investigate cancer metabolism.

## Approaches to assay cellular metabolism

The way cancer cells use metabolism to enable their pathological phenotypes is a key question that needs to be addressed. The techniques for assaying cellular metabolism and their application to cancer research have been extensively reviewed elsewhere (Jang et al., 2018; Kang et al., 2018; Kaushik and DeBerardinis, 2018); however, we briefly introduce some widely used techniques to facilitate the discussion on how these approaches can be applied to cancer models.

### Measurement of metabolite levels

One approach to investigate cellular metabolism is to measure the levels of intracellular metabolites (also referred to as ‘metabolite pool size’). To quantitatively assess total metabolite levels across experimental conditions, researchers can use a variety of chromatography–MS- or NMR-based analytical platforms. Depending on the approach, metabolite levels can be measured in a targeted (for a pre-determined set of metabolites) or untargeted manner, with a trade-off between the scope of detected metabolites and assay sensitivity (Jang et al., 2018; Kang et al., 2018). Furthermore, depending on the experimental set up, researchers can assess the relative or absolute levels of individual metabolites, with absolute quantitation requiring the use of purified standards (Jang et al., 2018; Kang et al., 2018). Relative quantitation is easier to accomplish and is thus most often used, particularly for untargeted metabolomics. However, an important consideration for relative metabolite quantification is that the absolute levels of the metabolites in the assayed sample will affect the interpretation of the relative change measured. That is, metabolites present at very low concentrations in the sample can exhibit large relative pool size changes in an experiment, despite these changes occurring over a concentration range that might be too low to have biological meaning. New approaches that help interpret the biological meaning of metabolite pool size changes, including metabolite activity screening and integration with other data such as transcriptional changes, have been developed and are reviewed elsewhere (Guijas et al., 2018; Jha et al., 2015; Forsberg et al., 2018). Conversely, when measuring absolute metabolite levels, the overall composition of the material being measured can give rise to matrix effects (Box 1) that can affect the apparent metabolite concentrations. This is particularly true in complex biological samples, where levels of some metabolites, such as phospholipids, either enhance or suppress the ionization of the metabolite of interest, leading to errors in quantitation (Trufelli et al., 2011). Analytical strategies, such as using isotopically labeled internal standards or changing how samples are prepared to include protein precipitation and/or phospholipid removal, have been developed to minimize the impact of matrix effects on metabolite quantitation (Bylda et al., 2014). Because metabolite pool sizes can be assessed across a variety of experimental samples – including cells, tissues, plasma and other biological fluids – metabolite level measurements have been increasingly applied to cancer research, regardless of how the metabolites themselves are measured.

Interpreting the biological meaning of metabolite level differences across conditions can be challenging, because measurements of metabolite pool sizes are static by nature. The observed differences in metabolite pool size between conditions could be caused by (1) changes in metabolite transport, (2) changes in metabolite production or (3) changes in metabolite consumption, and researchers need to conduct further experiments to understand why metabolite levels have altered (Chokkathukalam et al., 2014). Combining an assessment of metabolite pool size changes with metabolic enzyme expression changes can help provide mechanistic insight, and platforms such as XCMS Online (Forsberg et al., 2018) have been developed to assist with analysis. This multiomic approach has been utilized to infer why metabolite levels change in some contexts, including differentiation of macrophages into functionally discreet subsets (Jha et al., 2015).

### Extracellular flux measurements

Another method to assay cellular metabolism is to measure the consumption and release of nutrients, also referred to as the extracellular flux of nutrients. Extracellular flux analysis was used in the pioneering studies that determined the glycolytic nature of cancer, by measuring changes in glucose and lactate levels between the afferent and efferent circulation of a tumor (Cori and Cori, 1925). Currently, this analysis is more often performed in cultured cells, owing to the relative ease in sampling culture media over the course of an experiment. During an extracellular flux measurement, medium composition changes are evaluated over time, and consumption and production rates for various extracellular metabolites are calculated. This approach assesses which metabolites are net consumed or produced; however, this method does not assess how the metabolites are utilized. This caveat is important, as it has long been assumed that highly consumed nutrients such as glutamine and glucose are major contributors to cell mass in proliferating cells. In fact, much of the carbon from these molecules is excreted into the media of cultured cells, demonstrating that glutamine and glucose are less significant contributors to cell mass (Hosios et al., 2016). Despite these limitations, this technique has been successfully used to identify metabolic pathways important for cancer. For example, a comparison of extracellular fluxes with cellular proliferation rates using the National Cancer Institute panel of 60 human cancer cell lines (NCI-60) identified serine–glycine–one-carbon metabolism as a critical node for proliferating cancer cells (Jain et al., 2012). Additionally, extracellular flux measurements can be used along with stable isotope tracing (described below) to infer estimates of some intracellular fluxes (Antoniewicz, 2018; Niedenfuhr et al., 2015; Dai and Locasale, 2017). Thus, extracellular flux measurements can suggest hypotheses about metabolic alterations in cancer and can also complement other metabolic assays.

### Metabolite tracing using stable isotopes

Radioactive isotopic tracers (such as ^14^C or ^3^H) or stable isotope tracers (such as ^13^C or ^15^N) have long been used to study metabolism in microorganisms and the metabolic alterations associated with diabetes. These techniques allow for dynamic assessment of cellular metabolism outside of the static snapshot provided by the analysis of metabolite pool sizes. The various uses of isotopic tracers have been reviewed extensively elsewhere (Buescher et al., 2015; Kaushik and DeBerardinis, 2018; Jang et al., 2018; Kang et al., 2018; Antoniewicz, 2018; Niedenfuhr et al., 2015; Dai and Locasale, 2017). For stable isotope tracing, the model system is exposed to labeled substrates and the incorporation of these substrates into downstream molecules is measured to infer metabolic pathway usage. In this approach, the metabolic fluxes are not directly measured; however, some fluxes can be inferred based on characteristic isotopic label incorporation into specific metabolites (Buescher et al., 2015). Researchers can then estimate the absolute flux through metabolic pathways by combining isotope tracing with information from extracellular flux measurements and computational modeling (Antoniewicz, 2018; Niedenfuhr et al., 2015; Dai and Locasale, 2017). Thus, isotope tracing provides insight into the dynamic flux of nutrients through the metabolic network of cells, but requires the selection of an appropriate metabolic tracer and downstream analysis.

### Genetic or chemical interventions to study cellular metabolism

Indirect information about metabolic pathway use in cancer can be obtained from genetic approaches. In these assays, RNA interference (RNAi) or CRISPR-based pooled genetic screens (Box 1) interrogate the relevance of a given metabolic pathway for a cellular phenotype. RNAi-based genetic screening has identified the importance of mitochondrial oxidative phosphorylation (Birsoy et al., 2014) and one-carbon metabolism (Minton et al., 2018) for cancer cell growth under limiting glucose conditions, and the importance of dihydropyrimidine production for epithelial-to-mesenchymal transition (EMT) of cancer cells (Shaul et al., 2014). More recently, CRISPR-based genetic screening approaches have identified glutamic-oxaloacetic transaminase 1 (*GOT1*) as an essential gene for cancer cell proliferation under limiting mitochondrial electron transport chain function (Birsoy et al., 2015). CRISPR-based gene disruption has also been used to determine the functional consequences of simultaneously perturbing multiple metabolic enzymes including those involved in oxidative phosphorylation and the pentose phosphate pathway (Zhao et al., 2018).

In addition to genetic-based screening approaches, small molecule inhibitors, when available, can also be used to assess the functional requirements for a metabolic enzyme. A renewed interest in metabolism has sparked the development of metabolic enzyme inhibitors, and these inhibitors have been used to identify the molecular signatures of cancer cells that rely on specific metabolic pathways for proliferation. For example, pancreatic ductal adenocarcinoma cell lines were stratified based on their intracellular metabolite levels and subsequently treated with inhibitors based on this metabolic categorization. This process allowed for identification of cell lines that differentially respond to various metabolic inhibitors (Daemen et al., 2015). Furthermore, this approach has also been utilized in non-small cell lung cancer cell lines, which were found to have enhanced sensitivity to glutaminase (Box 1) inhibition if undergoing EMT (Ulanet et al., 2014). Mesenchymal cancer cell lines, as well those selected to be in a therapy-resistant state, have also been found to rely on redox and glutathione metabolism for survival (Viswanathan et al., 2017; Hangauer et al., 2017). The development of novel metabolic enzyme inhibitors has also led to new insights, suggesting that cancer cells in culture can adapt to metabolic pathway disruption via the use of alternative pathways (Pusapati et al., 2016; Boudreau et al., 2016; Biancur et al., 2017).

Functional metabolic studies using genomic or pharmacological interventions can be highly informative; however, extrapolating these results to understand how metabolism functions in a cell can still be challenging. For example, GOT1 essentiality induced by mitochondrial dysfunction does not indicate the directionality of flux through GOT1, or why the cell requires this enzyme, which is involved in multiple cell processes. Nevertheless, functional screening approaches hold promise for defining the metabolic requirements of cancer cells.

## What are current mammalian model systems and how can we test cellular metabolism in these systems?

**Fig. 1.**
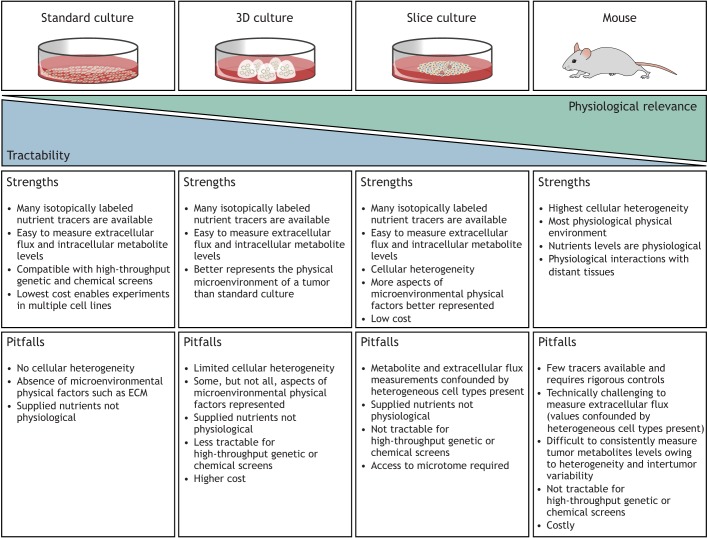
**Advantages and disadvantages of cancer model systems to study metabolism.** Cancer models vary in complexity from 2D monolayer cultures to *in vivo* systems. The most experimentally tractable cancer models are amenable to many different approaches to study metabolism, but fail to replicate the complex conditions found in a tumor. On the other hand, more physiologically complex cancer model systems, such as mouse models, are less tractable and complicate the interpretation of many metabolic assays. Models such as *ex vivo* cultures of cancer cells in 3D models like organoids or spheroids, or *ex vivo* cultures of tumor slices, lay on this continuum between tractability and physiological relevance. Each of these models presents with its own set of advantages and pitfalls that researchers need to consider when designing cancer metabolism experiments. ECM, extracellular matrix.

Assays of cellular metabolism are only one facet of what researchers need to study cancer metabolism. These assays must be deployed in appropriate disease model systems to gain insight into how metabolism is altered in cancer. Cancer models range from traditional monocultures to mouse cancer models to humans, and this section discusses how metabolic assays can be utilized in these systems. We highlight a key trade-off: with increasing complexity and physiological relevance of the model system, deriving insight from metabolism assays becomes more challenging (Fig. 1).

### Two-dimensional (2D) monocultures

Experiments using monocultures are fast, cost-sensitive, highly tractable and allow for the highest levels of stringency for experimental conditions (Fig. 1). Owing to easy access to both cells and extracellular media, all of the metabolic assays described above are feasible in 2D monocultures. Furthermore, the cost and scalability of monoculture allow for study of genetically diverse cell lines simultaneously (DeNicola et al., 2016; Jain et al., 2012; Timmerman et al., 2013; Kim et al., 2017; Sayin et al., 2017; Gross et al., 2014), for small molecule metabolic inhibitor screening (Daemen et al., 2015; Viswanathan et al., 2017; McMillan et al., 2018) and for the use of many labeled nutrient tracers (Jang et al., 2018). Scalability also enables pooled genetic screens, which require high representation of RNAi hairpins or CRISPR guide RNAs (Shalem et al., 2015). However, these model systems lack the cellular diversity observed in tumors, utilize media conditions that do not accurately mimic the conditions found in tumors, and the physical substrate upon which the cells grow is artificial. Thus, the metabolic phenotypes identified in culture can be different from the metabolism of cancers *in situ* (Fig. 1). Nevertheless, extensive insight has been gained from these models.

### Three-dimensional (3D) cultures

To better represent the physical environment of tumors, researchers can culture cancer cells in a matrix that allows 3D growth either as organoids or spheroids (Box 1). In this method, researchers seed cells in an extracellular matrix (ECM) for organoid cultures or grow cells as floating spheres (spheroids) in suspension. The resulting cells differ morphologically and physiologically from cells grown in monolayers (Breslin and O'Driscoll, 2013; Edmondson et al., 2014; Drost and Clevers, 2018). These culturing methods influence the spatial organization of cell surface receptors and induce physical constraints on cells, which cause changes in gene expression and cellular behavior (Edmondson et al., 2014). Organoid cultures can also incorporate more than one cell type (Ohlund et al., 2017). In some cases, 3D culture models have been able to better predict tumor drug responses *in vivo* (Vlachogiannis et al., 2018; Lee et al., 2018); however, 3D cultures still lack the cell and matrix diversity found within the tumor microenvironment. They also rely on media conditions that might not represent nutrient levels experienced by cells in tumors. With respect to modeling metabolism, it is not clear whether 3D models of cancer better predict the metabolism of cancer cells in tumors than do 2D monocultures, as evidence is currently lacking. In addition, although 3D cultures provide easy access to media and cells, allowing for extracellular flux, pool size and isotope tracing analyses, these models are less scalable than classical monocultures, which limits their use in small molecule or genetic screening experiments. Nevertheless, 3D models are still tractable to study the metabolism of cancer cells in a more relevant physical environment (discussed in the ‘Which tumor microenvironmental factors alter cancer metabolism?’ section).

### Tissue slice cultures

Cultured microtome (Box 1) slices of tissues and tumors have been used as a model of cancer that maintains cell diversity and tissue architecture. In fact, prior to the advent of monolayer cell culture, tumor slices were commonly used to study cellular metabolism (Warburg et al., 1927). In this model system, tissues are thinly sliced and placed in culture dishes. The thin slices allow oxygen and nutrients from the medium to reach the innermost layers of the tissue, while minimizing the proportion of damaged cells (Elliott, 1955). Because slice cultures allow access to media, extracellular flux measurements are possible, in addition to measuring pool sizes and tracing nutrient fate in the slice itself (Sellers et al., 2015; Fan et al., 2016). These models maintain the cellular heterogeneity observed in tumors and some aspects of the physical microenvironment, but it is important to note that the media used in tissue slice culture might not be representative of the nutrient microenvironment found in tumors *in vivo*. Furthermore, tissue slices are not genetically tractable and contain a heterogeneous mixture of metabolically active cells that contribute to metabolite level measurements. Despite their technical drawbacks, however, slice models provide a model to study how other cells and the physical microenvironment of tumors regulate cancer metabolism (discussed in the ‘Which tumor microenvironmental factors alter cancer metabolism?’ section).

### Mouse models of cancer

Animal models for studying cancer range from genetically engineered mouse models, allograft models and xenograft models derived from human tumors (Day et al., 2015; Gengenbacher et al., 2017). These *in vivo* models might better represent the physiological complexity and heterogeneity of cells observed in human cancers, and also allow interactions between the tumor and normal host tissues. However, when using mouse models to study metabolism, factors such as diet, strain, sex, age, husbandry and environmental stressors can affect metabolism and should thus be considered (Alquier and Poitout, 2018). Unfortunately, because the metabolism of the tumor is intertwined with the metabolism of the host animal, the ability to interpret many metabolic assays is limited. Intratumor metabolite levels can be measured, but as with culture slices, the observed changes in metabolite pool size are an average of the changes that occur across all the cells in the tissue, including noncancer cells (Reznik et al., 2018). Extracellular flux analysis of tumors is also challenging, as afferent and efferent blood vessels in rodent cancer models are not always accessible. It is possible to use stable isotope tracers via bolus injection(s) or continuous infusion of labeled nutrients into the circulation of tumor-bearing animals or patients (Sellers et al., 2015; Yuneva et al., 2012; Davidson et al., 2016; Hensley et al., 2016). However, interpretation of such labeling is complicated, as noncancer cells and tissues can rapidly consume some nutrients, and this can transfer the isotopic label from the supplied nutrient to intermediate metabolites that then supply the label indirectly to the cancer cells. Additionally, as with pool size measurements, both cancer and stromal cells contribute to labeling patterns in tumors. Lastly, functional assays of tumor metabolism are limited, as many inhibitors of metabolic enzymes cannot be used in animals owing to issues with bioavailability or toxicity, and functional genomics approaches are limited due to difficulty in maintaining RNAi or CRISPR library representation in tumor models (Doench, 2018; Gargiulo et al., 2014).

Thus, any model system utilized to study cancer metabolism has caveats that will affect the interpretation of the results (Fig. 1). Therefore, researchers must choose the system and metabolic assay that will appropriately address their specific experimental questions. Below, we discuss some recent experiments that assess the physiological relevance of metabolism in culture model systems in comparison to tumors *in vivo*.

## Do cancer cells adopt different metabolic behavior in different model systems?

While tissue culture models allow for tractable experiments to assess cellular metabolism, an important consideration is whether these model systems accurately model the disease in question (Wolpaw and Dang, 2018; Horvath et al., 2016). Thus, although some metabolic phenotypes of tumors, such as avid glucose uptake and lactate secretion, are maintained in culture models, several lines of evidence indicate that the microenvironment in which cancer cells grow can significantly affect cancer cell metabolism.

### Biochemical evidence suggesting that the environment alters metabolism

**Fig. 2.**
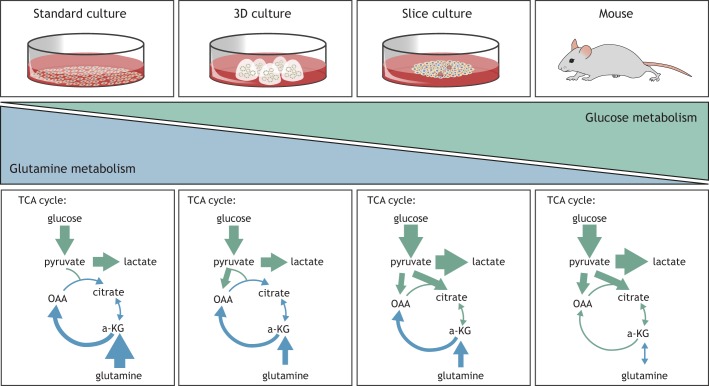
**The cancer model system chosen can affect metabolic phenotypes.** When the same cells are studied in different cancer model systems, the main carbon source that feeds the TCA cycle changes. For example, when cancer cells are implanted to form tumors in mice, an increase in the contribution of glucose-derived carbon to the TCA cycle, together with a reduced contribution of glutamine carbon, is observed, even though the same cells predominantly rely on glutamine in less physiological *in vitro* culture systems. a-KG, alpha-ketoglutarate; OAA, oxaloacetate; TCA, tricarboxylic acid.

Direct biochemical examination of central carbon metabolism using stable isotope-labeled nutrient tracers suggests that the tricarboxylic acid (TCA) cycle substrates can be utilized differently between tumors and 2D cultured cells. For example, in 2D culture models of lung cancer, glutamine is a primary carbon substrate for the TCA cycle, rendering glutaminase-mediated glutamine catabolism essential in most 2D culture settings (Davidson et al., 2016; Muir et al., 2017). In contrast, glutamine tracing experiments in lung tumors *in vivo* suggest only a minor contribution of glutamine to the TCA cycle (Davidson et al., 2016; Muir et al., 2017). Instead, lung tumors might favor using glucose for TCA cycle anaplerosis (Davidson et al., 2016; Sellers et al., 2015), and lung tumors are consequently sensitive to pyruvate carboxylase loss, an enzyme that allows glucose carbon to be used for TCA cycle anaplerosis (Fig. 2). Similarly, glioblastoma cells in culture depend on glutamine catabolism for TCA cycle anaplerosis and cell proliferation (Wise and Thompson, 2010; Tardito et al., 2015); however, tracing experiments in animals suggest that glioblastoma tumors utilize other TCA cycle substrates and can even net produce glutamine, arguing that these tumors are glutamine autonomous (Tardito et al., 2015; Marin-Valencia et al., 2012). These results suggest that tumors *in vivo* can show distinct nutrient labeling patterns that are different from what researchers observe in 2D culture models.

### Genetic experiments supporting the notion that microenvironment alters metabolism

Genetic experiments suggest that cancer cells have differential requirements for specific metabolic enzymes when cultured *in vitro* versus *in vivo*. Interestingly, genetic perturbations of some metabolic pathways can cause profound phenotypes *in vivo*, yet cause relatively minor phenotypes in culture and vice versa. Recycling metabolism such as autophagy or acetate recapture are striking examples of this phenomenon. Specifically, depletion of autophagy related 5 (*ATG5*) or autophagy related 7 (*ATG7*) (loss of autophagy), or acyl-CoA synthetase short chain family member 2 (*ACSS2*) (acetate recapture loss) is well tolerated by most cells in culture, but can dramatically decrease tumor growth *in vivo* (Amaravadi et al., 2016; Comerford et al., 2014; Schug et al., 2015). Furthermore, genetic screening experiments suggest extensive differences in metabolic pathway requirements between 2D culture models and tumors *in vivo*. RNAi and CRISPR screens to identify essential metabolic enzymes have been performed in both cell culture and in xenograft tumor models, and there was little concordance in the essential enzymes these screens identified (Yau et al., 2017; Alvarez et al., 2017; Possemato et al., 2011). While differences in oxygenation between *in vivo* tumors and culture models might drive some of these differences in metabolic pathway essentiality (Alvarez et al., 2017), it is likely that additional factors including the ECM, extracellular fluid and cell types present within the tumor microenvironment contribute to this differential metabolic pathway essentiality as detailed in the following section.

### Pharmacological evidence suggesting that the microenvironment alters cancer metabolism

Drugs targeting metabolic pathways also have different effects on cells in culture and in tumors. As discussed above, most cultured cells depend on glutamine catabolism, whereas many tumors formed from the same cells do not (Fig. 2). In line with these observations, CB-839, a drug targeting glutaminase, inhibits the proliferation of many cells in culture, but is less efficacious when the same cells are implanted to form tumors in animals (Davidson et al., 2016; Muir et al., 2017; Biancur et al., 2017). Similarly, drugs targeting mechanistic target of rapamycin (mTOR), a protein involved in nutrient sensing and metabolism regulation, are antiproliferative for many cancer cell lines, but have limited efficacy in treating mouse cancer models or patients (Palm et al., 2015). Drugs can also have antiproliferative effects against tumors that are not observed in culture models. For example, the antidiabetic drug metformin slows tumor proliferation (Morales and Morris, 2015), but supraphysiological doses (above those that can be achieved in tumors *in vivo*) are required to have antiproliferative activity in culture models (Gui et al., 2016).

Collectively, these studies argue that the tumor microenvironment alters cellular metabolism in a way that influences therapeutic response. This leads to a conundrum for researchers studying cancer metabolism. The most physiologically relevant models of cancer limit the experimental capacity to study metabolism, but experimentally tractable models do not accurately model all metabolic aspects of the disease (Fig. 1). Thus, understanding why culture models fail to recapitulate tumor metabolism will help researchers understand how the environment regulates cancer cell metabolism. In addition, identifying the environmental factors that regulate cancer cell metabolism could allow development of new culture models that are more predictive of the metabolic behavior of the tumor.

## Which tumor microenvironmental factors alter cancer metabolism?

As discussed above, the microenvironment in which cancer cells grow can significantly affect their metabolism. Here, we discuss the recent progress that has identified how some environmental factors can alter cancer cell metabolism.

### Nutrient levels alter cancer cell metabolism

Cells sense environmental nutrient levels via multiple nutrient-sensing mechanisms and adjust their metabolism accordingly (Palm and Thompson, 2017; Torrence and Manning, 2018; Efeyan et al., 2015), providing a basis for why the nonphysiological nutrient levels in standard culture media alter cancer cell metabolism. For example, oxygen is an important nutrient for cells, and low oxygen levels in tumors affect metabolism (Eales et al., 2016). Indeed, genetic screens suggest that differences in oxygen tension between tumors and standard culture models drive many different metabolic requirements of cancer cells (Alvarez et al., 2017; Keenan et al., 2015). Other nutrients have also been shown to regulate cancer metabolism. For example, high pyruvate levels found in cultured media blunt the antiproliferative effects of metformin, and culturing cells in media without pyruvate allows metformin to inhibit cell proliferation at concentrations that can be attained *in vivo* (Gui et al., 2016). Mechanistically, pyruvate allows cells to proliferate in the absence of a functional electron transport chain. Therefore, pyruvate alters redox metabolism and indirectly affects dependence on the electron transport chain, explaining why electron transport inhibitors like metformin (Wheaton et al., 2014) are less efficacious when pyruvate is available. Differences in pyruvate availability between organs *in vivo* can also affect cancer cell metabolism. Higher pyruvate availability in the lung microenvironment has been described as a factor that rewires breast cancer lung metastases to utilize pyruvate via the enzyme pyruvate carboxylase as a source of TCA cycle carbon (Christen et al., 2016). Thus, levels of pyruvate can be an important factor that regulates cancer cell metabolism.

The amino acid cystine is another example of how differences in nutrient levels between culture models and tumors alter cancer cell metabolism (Muir et al., 2017). High levels of cystine in standard culture media promote glutamine catabolism by increasing glutamate export through the cystine-glutamate antiporter solute carrier family 7 member 11 (*SLC7A11*). High glutamate export causes an increased reliance on glutamine catabolism in cultured cells. Thus, cystine indirectly affects glutamine catabolism via transport of glutamate, an intermediate in glutamine catabolism. Therefore, glutaminase inhibition has a greater antiproliferative effect *in vitro*, where cystine is abundant. Importantly, these examples illustrate that nutrient levels within the microenvironment alter metabolism in ways that might be distinct from the metabolic pathways in which these nutrients directly participate.

### Alternative nutrients present in the tumor microenvironment impact cancer cell metabolism

Although human plasma is estimated to contain ∼4600 circulating metabolites (Psychogios et al., 2011), only a small fraction of these are represented in standard culture media. Much of our understanding of cancer metabolism is limited to how cells engage with the limited set of substrates provided in standard media; however, the remaining ‘unexplored’ metabolites might also impact cellular metabolism. Recently, the metabolite itaconate, which can be produced by macrophages from TCA cycle intermediates (Strelko et al., 2011; Michelucci et al., 2013), was found to rewire metabolism by inhibiting succinate dehydrogenase (Lampropoulou et al., 2016) and inactivating vitamin B_12_ (Shen et al., 2017) in various immune cell types. Thus, in tumors, where itaconate can potentially accumulate owing to immune infiltration, itaconate levels can alter tumor metabolism. Additionally, itaconate affects cellular antioxidant metabolic programs via the activation of the transcription factor nuclear factor, erythroid 2 like 2 (*NRF2*; also known as *NFE2L2*), again potentially altering cancer cell metabolism (Bambouskova et al., 2018; Mills et al., 2018). In another example, growing leukemia cells in a culture medium containing nutrient levels found in the circulation, including many that are absent from standard culture media formulations, resulted in alterations in pyrimidine metabolism and in the response to 5-fluorouracil, a pyrimidine analog used as chemotherapy for several solid cancers (Cantor et al., 2017). Mechanistically, this effect was traced to the presence of uric acid, a nucleotide breakdown product present in human circulation that inhibits pyrimidine biosynthesis. Sulfur amino acid metabolism is also affected by metabolites that are not included in standard culture media. Circulating glutathione can be used by gamma-glutamyl transpeptidase (GGT)-positive tumors as a source of cysteine (Hanigan, 2014). In fact, GGT is a commonly elevated enzyme in tumors (Hanigan et al., 1999); however, the fact that glutathione is not included in standard culture media eliminates this potential source of sulfur amino acids for thiol metabolism when cells are studied *in vitro*.

Cancer cells in tumors are also exposed to metabolites that have traditionally been considered tumor metabolic ‘waste’ products, such as lactate (a glycolysis by-product) and ammonia (an amino acid catabolism by-product). Lactate and ammonia have long been considered to be metabolic waste products of tumors, as pioneering tumor metabolism studies found that nearly all tumors net secrete lactate (Cori and Cori, 1925; Warburg et al., 1927; Kallinowski et al., 1988; Gullino et al., 1967) and ammonia (Sauer et al., 1982). In these experiments, researchers measured metabolite levels in afferent arterial blood (entering the tumor) and in efferent venous blood (exiting the tumor). Lactate and ammonia levels were higher in the venous blood, indicating that nearly all tumors studied net produce and secrete these metabolites. As a result, there has generally been little interest in how cancer cells could utilize these metabolites. Recently, however, several groups have found that infusion of stable isotope-labeled lactate into tumor-bearing animals or human patients leads to substantial labeling of TCA cycle intermediates (Faubert et al., 2017; Hui et al., 2017), leading to the conclusion that tumors consume lactate as a carbon source. Similarly, tumor-bearing mice injected with isotopically labeled ammonia incorporate labeled nitrogen into tumor amino acids (Spinelli et al., 2017), thus leading to the conclusion that tumors can utilize ammonia. However, many metabolic reactions are rapidly reversible, allowing for cells to incorporate the isotopic label from a given metabolite even under conditions in which a metabolite is net secreted, a phenomenon termed exchange flux (Box 1) (Buescher et al., 2015; A. M. Hosios, Defining the contributors to mammalian cell mass, PhD thesis, Massachusetts Institute of Technology, 2017). For example, exchange between pyruvate and lactate occurs rapidly (Quek et al., 2016). Although these studies have argued that net consumption of lactate and ammonia can occur in some tumors (Faubert et al., 2017; Spinelli et al., 2017; Hui et al., 2017), experiments to measure tumor consumption or production of lactate and ammonia will provide insight into which subset of tumors consume or secrete these metabolites. Additionally, it is possible that a subpopulation of cancer cells within a tumor utilizes ‘waste’ metabolites generated from other parts of the tumor leading to intratumoral symbiosis. This has been proposed in the case of lactate (Doherty and Cleveland, 2013). Dissecting how subpopulations of cancer cells in tumors share metabolites will be informative in understanding tumor metabolism.

Beyond metabolites, macromolecules such as the ECM and circulating proteins are also present in the tumor microenvironment, but are not included in cell culture media, or present only at low levels. In some cases, cancer cells *in vivo* can consume and utilize these macromolecules as metabolic substrates. For example, pancreatic tumors acquire amino acids from circulating proteins via macropinocytosis (Box 1) (Davidson et al., 2017). Circulating proteins are not the only alternative nutrient source, as cancer cells have also been shown to acquire amino acids from the surrounding ECM proteins (Olivares et al., 2017; Muranen et al., 2017). Protein availability in the microenvironment can also influence which nutrient sources a cell uses to grow. Cells cultured in media with limiting amino acids can proliferate when supplied with physiological levels of the protein albumin, a condition that might better reflect the tumor microenvironment (Palm et al., 2015).

mTOR inactivation strongly inhibits cell proliferation in standard culture models. Surprisingly, when grown under low amino acid/high protein conditions, cells become resistant to mTOR inhibition. In fact, cells in low amino acid/high protein conditions grew better without mTOR activity, as mTOR inhibition either via Torin 1 treatment or via Raptor (also known as Rptor) depletion increased the autophagic flux required to process albumin (Palm et al., 2015). This result was recapitulated in mouse pancreatic cancer models, in which tumors paradoxically grew better upon mTOR inhibition. Thus, macromolecules such as proteins in the microenvironment might serve as alternative nutrient sources to support the metabolic pathways required for growth.

### Stromal cells can affect cancer metabolism

The cellular heterogeneity observed in tumors suggests that cancer cells exist in a metabolic community within the tumor microenvironment (Morandi et al., 2016; Lyssiotis and Kimmelman, 2017) that is not modeled in traditional cell cultures. One way in which stromal and cancer cells interact is via competition for limited nutrients. For example, cancer cells and infiltrating leukocytes both avidly consume glucose, which can become a limiting nutrient in the tumor microenvironment (Buck et al., 2017). Thus, cancer cells outcompete T-cells for glucose uptake, leading to decreased immune surveillance within the tumor (Ho et al., 2015; Chang et al., 2015). Cancer cells, by virtue of their high metabolic rate, not only compete with stromal cells for nutrients, but also condition the tumor microenvironment. For example, glucose-avid cancer cells not only compete with stromal cells for access to glucose, but also alter the tumor microenvironment by secreting lactate (Brand et al., 2016). Lactate in the microenvironment can, in turn, affect the behavior of stromal cells, such as infiltrating lymphocytes and macrophages, and can contribute to immunosuppression in tumors (Brand et al., 2016; Colegio et al., 2014).

Different cell populations within tumors can also engage in communal cell-cell nutrient sharing. Proper assessment of intercell nutrient sharing with current biochemical and metabolic tracing approaches is challenging, as these methods are unable to resolve net nutrient transfer versus exchange flux. Future studies that address nutrient sharing in co-cultures using chemical engineering-based analytical methods, such as performing metabolic flux analysis on co-cultures with appropriate tracers to infer metabolic phenotypes, or modeling techniques, such as using cell-type specific reporter proteins to assess cell-type specific labeling data, could help to unravel intercell nutrient sharing (Gebreselassie and Antoniewicz, 2015; Ghosh et al., 2014; Rossi et al., 2017; Ruhl et al., 2011; Shaikh et al., 2008). Thus, although the complex metabolic interactions between stromal and cancer cells have largely been unstudied, some insight has been gained into understanding cancer-stromal nutrient sharing in limited cases in which cancer cells are auxotrophic (Box 1) for a given nutrient and obtain that nutrient from other sources. For example, leukemia cells have limited ability to take up environmentally available cystine for cysteine metabolism, and therefore must rely on other environmental sources of cysteine, which are typically not supplied in standard culture media (Zhang et al., 2012). Under these conditions, leukemia cells rely on stromal cells to take up cystine and secrete reduced cysteine or glutathione, which can then be used as a source of cysteine. Thus, the presence of stromal cells alters cysteine and glutathione metabolism in cancer cells, which can, in turn, alter the sensitivity of these cells to chemotherapy. Specifically, glutathione can contribute to chemotherapeutic resistance, and thus stromal supply of cystine promotes leukemia survival upon drug treatment (Zhang et al., 2012). It is important to note that multiple cell types can interact in complex ways. For example, ovarian cancer cells similarly engage in commensal cystine metabolism with stromal fibroblasts, but tumor-infiltrating T-cells can compete for the stromal supply of cysteine (Wang et al., 2016).

Glutamine is another nutrient that is reported to be shared between stroma and cancer cells. Both glioblastoma and ovarian cancer cells in culture behave as glutamine auxotrophs, relying on an external supply of this nonessential amino acid (Yang et al., 2016; Wise and Thompson, 2010; Tardito et al., 2015). However, these tumors can synthesize their own glutamine and, in the case of glioblastoma, are reported to even net produce glutamine (Tardito et al., 2015; Marin-Valencia et al., 2012). Fibroblasts in ovarian cancer (Yang et al., 2016) and astrocytes in glioblastoma (Tardito et al., 2015) have subsequently been shown to locally produce glutamine from other substrates and supply tumor cells with this amino acid. Thus, stromal cells can supply nutrients to otherwise auxotrophic cancer cells, altering their metabolism and response to chemotherapy. However, the full extent to which cancer cells in tumors share their metabolism with neighboring cells will require development of new techniques to probe these intercellular fluxes.

Beyond nutrient sharing, stromal cells in the microenvironment can themselves serve as alternative sources of nutrients. For example, exosomes shed by fibroblasts contain metabolites that can sustain nutrient-starved cancer cells (Zhao et al., 2016). Stromal fibroblasts can also undergo autophagy to supply nutrients to cancer cells (Sousa et al., 2016). Stromal cells can even become nutrition for other cells via a process termed entosis (Box 1), which has been found to supply macrophages and cancer cells with nutrients in nutrient-deprived conditions (Hamann et al., 2017; Krajcovic et al., 2013).

### Physiochemical aspects of tumors can impact cancer metabolism

The tumor microenvironment also displays chemical and physical properties that are distinct from normal tissues and the circulation. Outside of animal models, cancer models commonly miss these aspects, which can play an important role in regulating cellular metabolism. For example, although it has long been appreciated that tumors are more acidic than the circulation (Tannock and Rotin, 1989), recent work suggests that environmental acidity profoundly affects metabolic gene expression (Chen et al., 2008), and leads to large changes in amino acid, fatty acid and antioxidant metabolism in cells (Lamonte et al., 2013; Corbet and Feron, 2017; Corbet et al., 2016). Additionally, large-scale genetic experiments in acidic media suggest different metabolic gene requirements in acidic conditions compared with standard culture media (Keenan et al., 2015). Thus, the chemical properties of the tumor environment affect cancer cell metabolism.

It is also increasingly apparent that the physical properties of the ECM within the tumor alter cancer cell metabolism (Tung et al., 2015; DelNero et al., 2018). Although it is unclear how the ECM modulates tumor metabolism, recent data suggest that the physical substrate upon which cancer cells grow contributes to differences in cancer cell metabolic phenotypes. Compared with cells growing as monolayers on stiff plastic, detached cells alter their glutamine (Jiang et al., 2016) and redox (Schafer et al., 2009) metabolism. In another example, breast cancer cells cultured in spheroid models show a dependence on proline catabolism that is not observed in standard 2D culture (Elia et al., 2017). These experiments argue that attachment to a physical substrate can affect metabolism. Furthermore, analysis of vascular cell metabolism on matrices of different stiffnesses suggests that matrix stiffness suppresses oxidative phosphorylation while activating glutamine catabolism (Bertero et al., 2016). Thus, additional studies are needed to elucidate how the physical properties of the tumor influence cellular metabolism and the extent to which the ECM explains differences in metabolism between tumors and cultured cancer cells.

## Conclusions and future directions

As discussed in this article, various components of the tumor microenvironment affect the metabolic behavior of cancer cells, and thus noncell-autonomous factors play a major role in dictating cellular metabolism. Importantly, this affects cancer cell response to some therapies, including those that target metabolism (Zhang et al., 2012; Muir et al., 2017; Palm et al., 2015; Cantor et al., 2017). Thus, it is important to consider these factors when studying cancer metabolism, which might not always be modeled in traditional cell culture. Animal cancer models provide an alternative, but have limited experimental tractability to probe metabolism. We suggest that these issues could be addressed by developing new techniques to better study cancer cell metabolism *in vivo*, and by better characterizing the tumor microenvironment to design new *ex vivo* models of cancer that better represent the tumor microenvironment and remain experimentally tractable.

Towards the first goal, emerging technologies such as imaging MS will allow spatial examination of metabolite levels in tumors (Aichler and Walch, 2015; Spengler, 2015), and could begin to resolve metabolite differences between stromal and cancer cells in tumors. New theoretical and computational tools to measure intercell metabolite sharing between different cell types using stable isotope tracing (Gebreselassie and Antoniewicz, 2015; Ghosh et al., 2014; Rossi et al., 2017; Ruhl et al., 2011; Shaikh et al., 2008) might also facilitate the study of metabolic commensalism in tumors. New developments in CRISPR-based genetic screening approaches could make the functional screening of metabolic pathway requirements possible in mouse cancer models (Michlits et al., 2017; Chow and Chen, 2018; Schmierer et al., 2017). Furthermore, revisiting old techniques with new analytical technologies could also provide new insight into tumor metabolism. For example, extracellular fluxes of many metabolites could be quantitated in tumors by using new analytical platforms coupled with existing techniques to isolate tumor afferent and efferent blood.

Toward the second goal, a better understanding of how microenvironmental variables influence tumor metabolism is needed. There is increasing knowledge of which cell populations are present in a tumor (Lee et al., 2017; Heindl et al., 2015; Yuan, 2016; Carmona-Fontaine et al., 2017) and the physical matrix in which these cells reside (Naba et al., 2017; Pearce et al., 2018). However, less is known about the soluble environment of tumors, such as the nutrients that are present in the extracellular compartment within tumors. Combining old techniques with new analytical capabilities could again prove useful. Methods for harvesting the interstitial fluid from tissue and tumors have long existed (Wiig and Swartz, 2012). Combining these methods with new MS-based techniques to measure metabolite levels will provide new insight into the ‘nutritional microenvironment’ of tumors. New tumor-on-a-chip microfluidic devices (Sleeboom et al., 2018) will also make incorporating newly emerging microenvironmental information into *ex vivo* cancer models possible. We anticipate that this will provide new insight into the metabolism of cancer and stromal cells in tumors, and the microenvironmental variables that constrain metabolism. This information will ultimately provide insight into the metabolic processes that should be targeted for cancer therapy and the contexts in which the therapies will be most efficacious.

This article is part of a special subject collection ‘Cancer metabolism: models, mechanisms and targets’, which was launched in a dedicated issue guest edited by Almut Schulze and Mariia Yuneva. See related articles in this collection at http://dmm.biologists.org/collection/cancermetabolism.
